# The Relationship Between Dietary and Supplemental omega-3 Highly Unsaturated Fatty Acid Intake, Blood and Tissue omega-3 Highly Unsaturated Fatty Acid Concentrations, and Colorectal Polyp Recurrence: A Secondary Analysis of the seAFOod Polyp Prevention Trial

**DOI:** 10.1016/j.tjnut.2024.12.004

**Published:** 2024-12-31

**Authors:** Ge Sun, Harriett Fuller, Hayley Fenton, Amanda D Race, Amy Downing, Colin J Rees, Louise C Brown, Paul M Loadman, Elizabeth A Williams, Mark A Hull

**Affiliations:** 1 Leeds Institute of Medical Research, University of Leeds, Leeds, United Kingdom; 2 Institute of Cancer Therapeutics, University of Bradford, Bradford, United Kingdom; 3 Population Health Sciences Institute, Newcastle University, Newcastle upon Tyne, United Kingdom; 4 MRC Clinical Trials Unit at University College, London, United Kingdom; 5 School of Medicine and Population Health, University of Sheffield, Sheffield, United Kingdom

**Keywords:** colorectal cancer, compliance, diet, docosahexaenoic acid, eicosapentaenoic acid

## Abstract

**Background:**

The seAFOod randomized controlled trial tested colorectal polyp prevention by the omega-3 (ω-3) highly unsaturated fatty acid (HUFA) eicosapentaenoic acid (EPA) and aspirin. Variable dietary intake of omega-3 HUFAs (also including docosahexaenoic acid [DHA]) and differential EPA capsule compliance could confound analysis of trial outcomes.

**Objective:**

The objective of this study was to investigate the relationship between *total* (diet and capsule) daily omega-3 HUFA intake, red blood cell (RBC), and rectal mucosa omega-3 HUFA concentrations, and colorectal polyp outcomes in a secondary analysis of the seAFOod trial.

**Methods:**

Individual-participant dietary omega-3 HUFA intake (mg/d) was derived from food frequency questionnaires using the European Prospective Investigation into Cancer and Nutrition-Norfolk fatty acid nutrient database. Capsule EPA intake (mg/d) was adjusted for compliance (capsule counting). Fatty acids were analyzed by liquid chromatography-tandem mass spectrometry (as % of total fatty acids). HUFA oxidation was measured using the HUFA/saturated fatty acid (SAT) ratio. The colorectal polyp detection rate (PDR; % with ≥1 polyps) and polyp number per participant were analyzed according to the change in RBC EPA concentrations during the trial (ΔEPA), irrespective of treatment allocation.

**Results:**

There was a small degree of HUFA degradation over time in RBC samples stored at > −80^o^C at research sites (*r* = −0.36, *P*<0.001 for HUFA/SAT ratio over time), which did not affect analysis of omega-3 HUFA concentrations. Low baseline EPA concentration, as well as allocation to EPA and % compliance, were associated with a high ΔEPA. Individuals with a ΔEPA value >+0.5% points (ΔEPA_high_), irrespective of allocation to EPA or placebo, had a lower PDR than ΔEPA_low_ individuals (odds ratio: 0.63; 95% confidence interval [CI]: 0.40, 1.01) and reduced colorectal polyp number (incidence rate ratio: 0.74; 95% CI: 0.54, 1.02).

**Conclusions:**

Analysis of the seAFOod trial according to the change in EPA concentration, instead of treatment allocation, revealed a protective effect of EPA treatment on colorectal polyp recurrence (ISRCTN05926847).

## Introduction

The seAFOod polyp prevention trial was a double-blind, randomized, placebo-controlled 2 × 2 factorial trial that tested the efficacy of the omega-3 highly unsaturated fatty acid (HUFA), C20:5*n*–3 EPA, and aspirin for prevention of colorectal polyps in “high risk” individuals undergoing colonoscopy surveillance after removal of multiple colorectal polyps [1]. There was no significant effect of EPA-free fatty acid 2000 mg or aspirin 300 mg taken daily for 12 months on the primary trial endpoint, which was the percentage of individuals with ≥1 colorectal polyps [the polyp detection rate (PDR)] at surveillance colonoscopy [1].

Participants who were allocated active omega-3 HUFA capsules received either 2000 mg >99% pure EPA fatty acid (FFA) or the FFA equivalent provided as 2780 mg 90% EPA triglyceride (TG) for approximately one-third of participants (see the primary trial publications for a detailed description of the Investigational Medicinal Product [IMP] switch when supply of the former formulation ceased [1,2]). In both cases, identical placebo capsules contained mixed capric and capryllic acid medium-chain TGs [2]. Participants were instructed not to take any fish oil or cod-liver oil supplements during the trial, but no specific dietary advice was provided. Therefore, differential dietary intake of marine-derived, bioactive omega-3 HUFAs (EPA and C22:6*n*–3 DHA) by seAFOod trial participants could have confounded trial outcomes. In the primary seAFOod trial report, we confirmed that oily and total fish intake did not change significantly at treatment group level during trial participation [1]. However, proof that fish intake was stable during the trial (thereby excluding an overall increase in dietary omega-3 HUFA intake driven by trial participation) does not address whether individual participant-level dietary marine-derived omega-3 HUFA intake might confound the comparison of trial colonoscopy outcomes between groups receiving either placebo or active EPA supplementation. The importance of measuring blood omega-3 HUFA concentrations in clinical omega-3 HUFA intervention studies in order to understand other factors, including diet, that affect blood omega-3 HUFA concentrations and trial outcomes has previously been highlighted [3].

In this secondary analysis of the seAFOod trial, we determined the total daily omega-3 HUFA (EPA+DHA) intake for individual seAFOod trial participants as the combination of dietary intake (primarily from fish and other seafood) derived from food frequency questionnaire (FFQ) data and intake from the capsule intervention (corrected for individual compliance). We aimed to investigate the relationship between total omega-3 HUFA intake and blood/rectal mucosal concentrations of EPA and DHA. We then performed an exploratory analysis of seAFOod trial colorectal polyp outcomes according to the change in red blood cell (RBC) EPA concentration during the trial intervention rather than by stratification to placebo or EPA as per the original randomized trial design.

## Methods

### Study approval and registration

This project was a component of the STOP-ADENOMA study, which obtained approval from the London and Surrey Borders Research Ethics Committee (19/LO/1655) and is registered as ISRCTN05926847.

### Calculation of marine omega-3 PUFA intake

seAFOod trial participants completed a European Prospective Investigation into Cancer and Nutrition (EPIC) short-form FFQ at baseline and at the exit trial visit after the 12-mo intervention period [1,2]. Dietary fish intake was converted into EPA and DHA intake (mg) per day using the EPIC-Norfolk fatty acid nutrient database [4].

Capsule IMP compliance was measured by capsule counting at trial assessments at 6 and 12 mo when participants returned unused capsules [2]. Participants were scheduled to take either 4 capsules daily (if receiving EPA-FFA) or 5 capsules daily (if receiving EPA-TG). Each participant only received a single EPA formulation during the trial intervention phase. The total number of returned capsules during the trial intervention period was determined, and the percentage capsule compliance was derived for each participant as (number of capsules expected to be taken between receiving IMP and the day before trial exit colonoscopy) minus [total number of capsules returned (adjusted for the excess capsules provided to each participant)]/(number of capsules expected to be taken between receiving IMP and the day before trial exit colonoscopy). The expected number of capsules taken was adjusted for periods when the IMP was stopped (e.g., before an invasive procedure) or the dose was reduced per-protocol (driven by gastrointestinal adverse reactions) [2]. The effective daily dose of supplemental EPA in mg FFA equivalents for each trial participant allocated to active IMP was derived by multiplying the percentage compliance value by 2000 mg. This value was 0 for participants that were allocated to placebo capsules.

Daily total EPA+DHA intake was calculated for each participant by summating supplemental EPA intake and dietary EPA+DHA intake.

### Measurement of HUFA concentrations in RBCs and rectal mucosa

Blood and rectal mucosal sampling during the seAFOod trial has been described in detail [1,2]. Blood sampling was scheduled at trial entry [baseline, before IMP was started; visit (V) 1], after 6 mo treatment (V4), and at the end of the 12-mo intervention period just before colonoscopy (V6). Biopsies of normal rectal mucosa were obtained at the exit colonoscopy at V6 [1,2]. Blood samples were collected in ethylenediaminetetra-acetic acid and immediately centrifuged at 4^o^C to separate RBCs, the buffy coat, and plasma fraction into 500 μl aliquots in 2 ml cryovials. The seAFOod trial involved >50 hospital endoscopy units during the trial intervention phase between November 2011 and June 2017. Many smaller hospital units did not have access to −80^o^C freezer facilities. Therefore, local storage of blood fractions and rectal mucosa ranged from −80^o^C to −20^o^C for a variable amount of time prior to courier transport on dry ice to the central seAFOod trial biobank (University of Bradford) for subsequent storage at −80^o^C. All research sites were required to record the storage temperature of each blood, urine, and rectal mucosal sample.

Nine fatty acids, including EPA, C20:4*n*–6 arachidonic acid (AA), C22:5*n*–3 DPA, and DHA, were quantified using LC-MS/MS as described [5]. In brief, fatty acids were extracted from washed RBC membranes or rectal mucosal homogenates by isopropanol/chloroform extraction, with deuterated *α*-linolenic acid (ALA)-d_14_ as an internal standard. Sample supernatants were completely evaporated, reconstituted in acetonitrile, and then subjected to acidic saponification with 5 M hydrochloric acid at 80^o^C [5]. Following neutralization with sodium hydroxide and a second evaporation step, extracted fatty acids were derivatized using 4-[2-(*N*,*N*-dimethylamino)ethylaminosulfonyl]-7-(2-aminoethylamino)-2,1,3-benzoxadiazole [5]. Analysis was performed using a Waters Alliance 2695 High Pressure LC system in combination with a Waters Micromass Quattro Ultima triple quadrupole mass spectrometer operated in electrospray ionization positive MRM mode. Analyte separation was achieved by a gradient LC method using a HiChrom RPB column (2.1 mm × 250 mm, 5 μm) and the following mobile phase constituents: ultra-pure water, methanol, and formic acid [5]. Data are expressed as the percentage of each fatty acid relative to the total fatty acid peak chromatographic area. The ratio of measured HUFAs (EPA + AA + *n–*3DPA + DHA) to saturated fatty acids (SAT; C16:0 palmitic acid + C18:0 stearic acid) was used as a biomarker of *ex vivo* fatty acid oxidation [6].

### Colorectal polyp outcomes in the seAFOod trial

The primary outcome of the seAFOod polyp prevention trial was the polyp (previously termed “adenoma) detection rate (PDR), which is the percentage of individuals with ≥1 polyps (including both adenomatous and serrated polyps) at surveillance colonoscopy 12 mo after clearance screening colonoscopy [1]. It is now recognized that the secondary endpoint of colorectal polyp number is a better readout of polyp prevention efficacy in high-risk populations with multiple polyps [7,8]. Consequently, we have since used polyp multiplicity, in parallel with the PDR, in secondary seAFOod trial outcomes analyses [7,9].

### Statistical analysis

The percentage omega-3 HUFA content of RBCs and rectal mucosa (EPA, DHA, and EPA+DHA) is presented as the mean value ± SD or as the median value and the IQR, depending on the distribution of individual values.

The relationship between omega-3 HUFA intake and omega-3 HUFA concentrations in RBCs and rectal mucosa was investigated by Spearman or Pearson correlation, depending on the data distribution. Continuous variables were compared using either the Wilcoxon rank sum test or t-test. For multiple comparisons, post hoc analysis with Bonferroni correction was applied.

In order to identify factors associated with baseline RBC omega-3 HUFA concentrations and omega-3 HUFA concentrations in rectal mucosa, univariate linear regression analysis was conducted, followed by multivariate linear regression analysis adjusted for confounders that could affect the relationship between omega-3 HUFA intake and concentrations (BMI, tobacco smoking, and RBC sample storage conditions at research sites). According to the seAFOod trial protocol [1,2], rectal mucosa was obtained only at the colonoscopy performed at the end of the intervention period. Therefore, analysis of factors, including dietary intake of omega-3 HUFAs, associated with rectal mucosal omega-3 HUFA concentrations was restricted to participants randomly assigned to placebos only in order to avoid potential confounding of results by EPA and aspirin treatment. All other analyses of baseline and postintervention omega-3 HUFA concentrations considered the whole seAFOod trial cohort with available data, including both EPA and placebo users.

A similar multivariate linear regression model was developed to investigate factors associated with the change in individual RBC % EPA concentration (ΔEPA) during the trial intervention period between the baseline (V1) measurement and the respective value at 6 mo (V4). This model included sex, allocation to active EPA capsules and % capsule compliance, and allocation to aspirin treatment, as well as the baseline RBC % EPA value.

Consistent with prior seAFOod trial analyses [7,9], negative binomial regression was used to investigate the influence of different factors on colorectal polyp number due to the overdispersion and positive-skewed distribution of colorectal polyp number data [2]. Logistic regression analysis was conducted to investigate factors governing the PDR [7,9]. Consistent with our other secondary trial analyses, both multivariate models included male sex, aspirin use, and repeat colonoscopy at trial entry as variables, as well as the ΔEPA value [7,9].

All analysis was conducted using R Studio, version 2021.09.0.

## Results

At seAFOod trial randomization, 624 (88% of the 707 randomly assigned participants who provided postrandomization trial outcome data) patients provided a blood sample, and 649 (92%) completed a baseline FFQ. At the end of the 12-mo intervention period, 522 (74%) participants provided a blood sample, and rectal mucosa was obtained from 519 (73%) participants at the trial exit colonoscopy. The number of participants with available omega-3 HUFA and FFQ data in each of the 4 treatment groups is reported in Supplemental Table 1. There was no significant difference in demographic characteristics between seAFOod trial participants included and not included in this study [10].

### RBC sample storage and RBC HUFA concentrations

**FIGURE 1:**
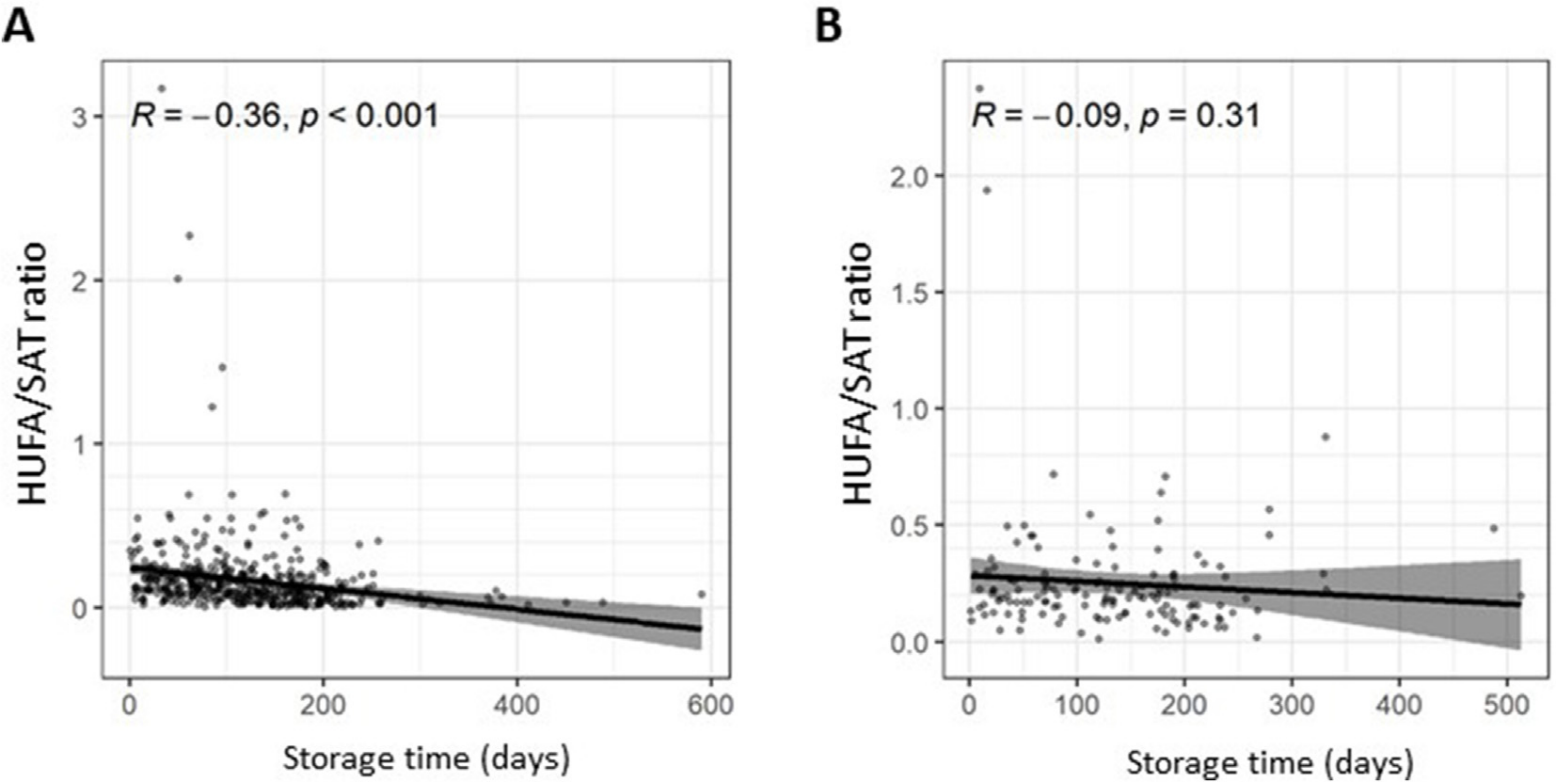
The ratio of HUFAs to saturated fatty acids (HUFA/SAT ratio) in baseline RBC samples from seAFOod trial participants according to the duration of storage at trial research sites at either A) >−80^o^C, or B) −80^o^C. The HUFA/SAT ratio was calculated as the ratio of the sum of % content values for EPA, AA, *n–*3DPA and DHA divided by the sum of the % amounts of palmitic acid and stearic acid. Individual data points represent the HUFA/SAT ratio for the baseline RBC sample for every seAFOod trial participant who provided a baseline blood sample regardless of subsequent treatment allocation. Of 463 V1 samples stored at a temperature greater than **−**80^o^C, 333 (72%) samples were stored at **−**20^o^C, 53 (11%) samples were stored at a temperature between **−**21^o^C and **−**30^o^C, and 77 (17%) samples were stored at a temperature between **−**31^o^C and **−**79^o^C. Abbreviations: HUFA, highly unsaturated fatty acid; RBC, red blood cells.

Storage of RBCs at temperatures above −80^o^C is associated with loss of HUFAs over time, which is believed to be driven by oxidation in the presence of heme iron [6,[11], [12], [13], [14], [15]]. The reported rate of HUFA loss over time varies depending on several factors, including RBC aliquot volume and presence of an antioxidant [6,[11], [12], [13]]. Given practical and financial constraints at research sites, which limited −80^o^C storage capability and weekly temperature-controlled sample transfer, we adopted a pragmatic approach by collecting a large volume RBC aliquot (500 μl) in a sealed cryovial and coordinating sample transfer from several research sites to the central −80^o^C trial biobank at the same time. Therefore, RBC samples were stored for a variable duration and at different temperatures (Supplemental Figure 1), allowing us to investigate the effect of temperature (−80^o^C, n = 142 compared with >−80^o^C, n = 463) and storage time [−80^o^C, median 128 d (range: 56–184 d); >−80^o^C, median 117 d (range: 65–170 d) on the HUFA/SAT ratio of baseline RBC samples from the whole trial cohort before the trial intervention was given. RBC samples stored at temperatures >−80^o^C before transfer to the central trial biobank displayed a reduction in HUFA/SAT ratio over time (*r*=−0.36, *P*<0.001; Figure 1A), unlike samples that were stored at the research site at minus 80^o^C (*r*=−0.09, *P*=0.31; Figure 1B). In the absence of longitudinal measurements on the same samples, calculation of the rate of loss of HUFAs was not possible. However, a comparison of patient samples stored at the research site for different periods of time did not suggest substantial HUFA loss even ≤200 d (Figure 1A and B). The proportion of all RBC samples taken at V1, V4, and V6 that were stored at either −80^o^C or greater than −80^o^C was similar across all trial treatment groups (Supplemental Table 2). Consistent with HUFA loss over time at a storage temperature >−80^o^C driven by heme iron, there was no reduction in the HUFA/SAT ratio of rectal mucosa over time, even if stored at >−80^o^C before transfer to the central biobank (*r*=−0.01, *P*=0.97; Supplemental Figure 2).

### Dietary omega-3 HUFA intake and RBC concentrations before the trial intervention

**FIGURE 2:**
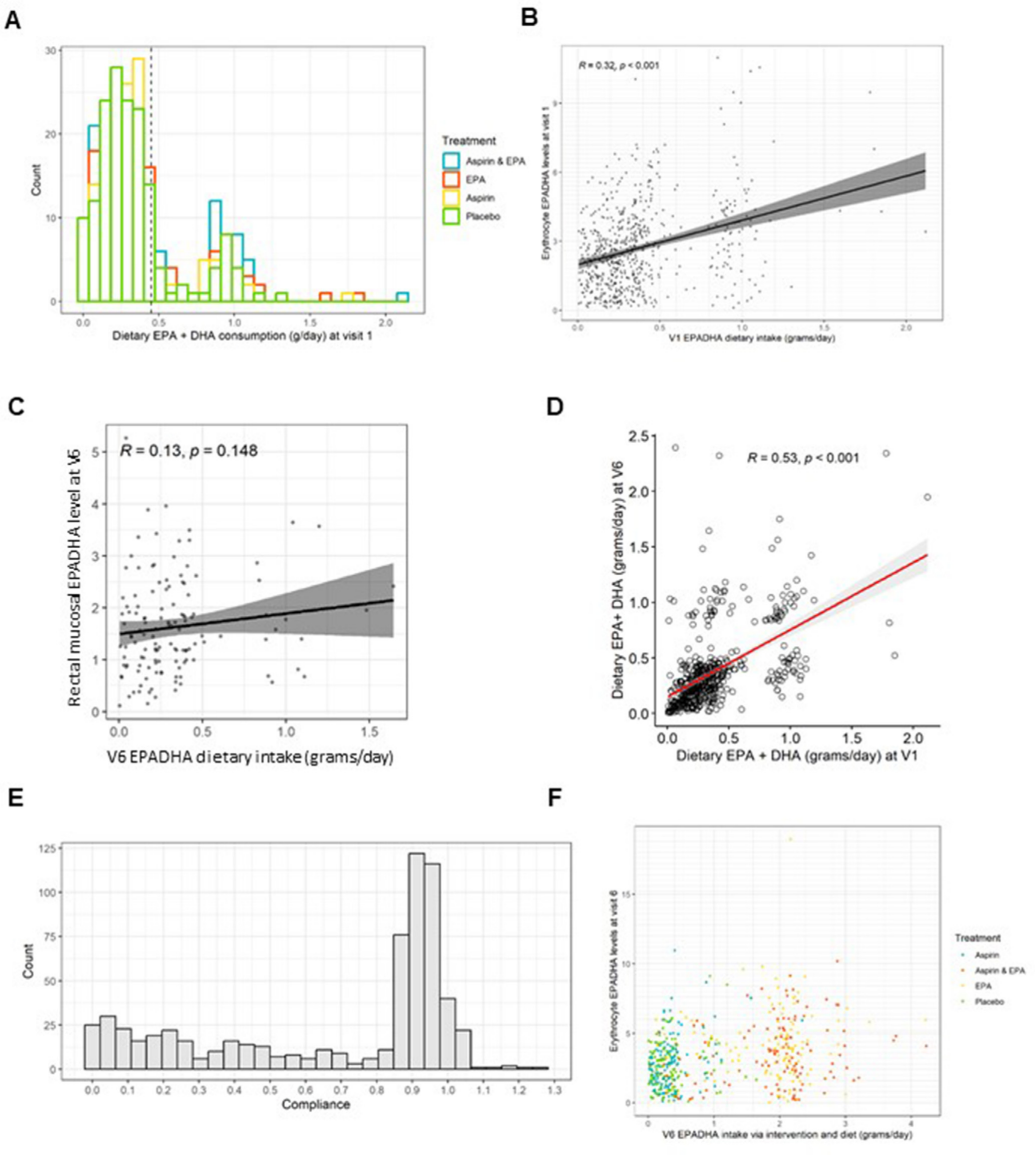
Dietary and supplement consumption of omega-3 PUFAs in seAFOod trial participants, according to FFQ and capsule IMP compliance, in relation to RBC omega-3 PUFA concentrations. (A) Overlaid frequency histogram of daily dietary EPA+DHA intake categories in the 4 treatment groups. The dashed line represents the dietary EPA+DHA intake per day (0.45 g/d) equivalent to the UK Government recommendation to eat 2 portions of fish per week, 1 of which should be oily [16]. (B) The relationship between daily dietary EPA+DHA intake and the RBC EPA+DHA concentration at baseline (V1). (C) The relationship between daily dietary EPA+DHA intake measured by the V6 FFQ and the rectal mucosal EPA+DHA concentration at colonoscopy (V6) in participants allocated to placebos only. (D) The relationship between daily dietary EPA+DHA intake at baseline (V1) and at the end of the trial intervention (V6). (E) Profile of IMP capsule compliance in seAFOod trial participants. Percentage compliance across the whole intervention period is expressed as a fraction on the X axis. (F) The relationship between overall EPA+DHA intake (from diet and supplement) and the individual RBC EPA+DHA concentration stratified by treatment arm. Abbreviations: FFQ, food frequency questionnaire; UK, United Kingdom; IMP, Investigational Medicinal Product.

We investigated the relationship between dietary EPA+DHA intake and RBC concentrations in seAFOod trial participants before IMP was started. Figure 2A shows that dietary EPA+DHA intake values displayed a bimodal distribution, including a majority (*n* = 510; 77%) of participants who consumed less than the daily intake of EPA+DHA (∼0.45 g/d) recommended by several national dietary guidelines, which advise eating 2 portions of fish per week, 1 of which should be oily [16,17], but also a number of individuals (*n* = 89; 13%) who were consuming between 0.8 and 1.2 g/d of dietary EPA+DHA. The distribution of daily intake values was comparable across the 4 intervention groups, as would be expected from trial randomization (Figure 2A). There was a moderate strength (*R*=0.32), statistically significant (*P*<0.001) correlation between dietary EPA+DHA intake and the combined EPA+DHA concentration in RBCs at baseline, consistent with previous studies (Figure 2B) [[18], [19], [20]]. Several other clinical factors also influence circulating blood omega-3 HUFA concentrations, including sex, body weight, alcohol intake, and tobacco smoking [20]. In univariate analyses, female sex and increasing alcohol intake were associated with higher RBC EPA concentrations at baseline, whereas BMI and tobacco smoking displayed an inverse relationship with RBC EPA+DHA concentrations (Supplemental Table 3). A multivariate linear regression model including BMI and tobacco smoking, as well as an interaction term for duration of RBC sample storage at the research site and a storage temperature of −80^o^C or greater, confirmed the strong association between dietary EPA+DHA intake and the RBC EPA+DHA concentration [β-coefficient: 1.71 (95% CI: 1.28, 2.13); *P*<0.001; Table 1]. The model also indicated a small effect on the duration of RBC sample storage at the research site even at temperatures >−80^o^C (β-coefficient: −0.008 [95% CI: −0.009, −0.006]; *P*<0.001; Table 1).

**TABLE 1:** Multivariate model of factors related to the baseline % RBC EPA concentration in seAFOod trial participants.

Baseline EPA concentration	Effect estimate	95% confidence limits	t value	*P* value
Intercept	2.71	2.23, 3.19	11.08	<0.0001
Dietary EPA+DHA intake	1.71	1.28, 2.13	7.92	<0.0001
Storage at >−80^o^C × storage time	**−**0.008	**−**0.009, **−**0.006	**−**8.51	<0.0001
Storage at −80^o^C × storage time	0.002	**−**0.0002, 0.004	1.82	0.07
Never tobacco smoker	0.57	0.17, 0.98	2.78	0.006
Previous tobacco smoker	0.43	0.04, 0.81	2.18	0.03
BMI obese (≥30 kg/m^2^)	**−**0.61	**−**0.98, **−**0.23	**−**3.15	0.002
BMI overweight (25–29.9 kg/m^2^)	**−**0.38	**−**0.74, **−**0.01	**−**2.02	0.04
BMI normal/underweight (<25 kg/m^2^)	**−**1.11	**−**3.35, 1.13	**−**0.98	0.33

Abbreviation: RBC, red blood cells.

### Dietary omega-3 HUFA intake and rectal mucosal omega-3 HUFA content

The % EPA content of RBC membranes and rectal mucosa was similar (Supplemental Table 3 and Supplemental Table 4). However, the relative DHA content of rectal mucosa was lower than that of RBC membranes (Supplemental Table 3 and Supplemental Table 4). In contrast to the RBC omega-3 HUFA data, there was only a weak correlation between dietary EPA+DHA intake and rectal mucosal EPA+DHA content (*R*=0.13), which did not reach statistical significance (*P* = 0.15; Figure 2C). The clinical factors that predicted RBC EPA+DHA concentrations in seAFOod participants at baseline (Supplemental Table 3) did not demonstrate an association with rectal mucosal EPA+DHA concentrations (Supplemental Table 4).

### Overall omega-3 HUFA intake during the intervention phase of the seAFOod trial

In the primary analysis of the seAFOod trial, we compared total fish and oily fish intake recorded by the baseline FFQ and the FFQ completed at the end of the intervention period at trial treatment arm concentration [1,2]. It was reported that there was no clear increase in fish intake after joining the trial and no imbalance between treatment groups that might confound trial outcomes according to treatment allocation [1,2]. Here, we compared dietary EPA+DHA intake at baseline with that recorded at the end of the intervention at 12 mo at individual trial participant level. The mean change in dietary EPA+DHA intake between the baseline FFQ and corresponding FFQ completed at the end of the trial intervention period was <0.01 (SD 0.31) g/d, with a moderate strength correlation (*r* = 0.53, *P* < 0.001; Figure 2D). These data suggest stable individual dietary marine-derived omega-3 HUFA intake across the entire cohort during the trial intervention phase.

Although overall compliance with capsule IMP during the seAFOod trial was excellent (mean 95%) [1,2], individual variability in % compliance values for EPA capsule use was evident (IQR: 27%–94%; Figure 2E). Therefore, in order to calculate the total daily intake of omega-3 HUFAs during the trial intervention period, we corrected the daily EPA supplement intake for each participant’s % compliance value (2000 mg EPA-FFA × % compliance value/100) before adding it to the daily dietary EPA+DHA intake value for each participant (derived from the end of intervention FFQ).

### Distribution of RBC EPA+DHA concentrations during the seAFOod trial intervention period

The distribution of values for the total daily intake of marine-derived omega-3 PUFAs (diet plus supplemental EPA) and RBC EPA+DHA concentrations according to the treatment group to which a trial participant was randomly assigned is described in Figure 2F. There was clear separation of individual daily EPA+DHA intake values between EPA-containing and no-EPA treatment arms consistent with the major contribution of supplemental EPA intake in individuals in treatment arms containing EPA compared with dietary omega-3 HUFA intake (Figure 2F). However, there was appreciable overlap of trial participants (at ∼1 g EPA+DHA daily intake) who either received placebo but had a relatively high dietary intake or who had poor compliance with EPA capsules (Figure 2F).

**FIGURE 3:**
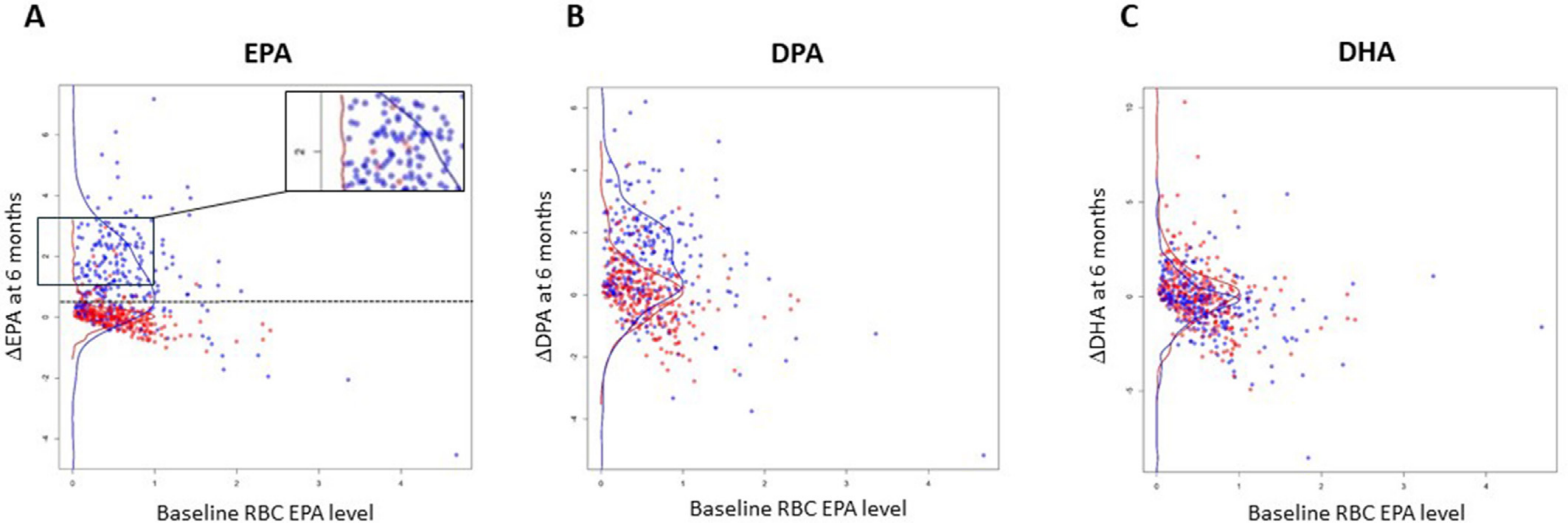
The change in RBC concentrations of EPA, DPA and DHA at 6 mo during seAFOod trial participation according to random assignment to EPA or placebo. The difference in omega-3 HUFA concentration after treatment for 6 mo (V4) compared with baseline (V1) (ΔEPA) is plotted at individual participant-concentration according to the baseline EPA value for (A) EPA, (B) DPA, and (C) DHA. Summary curves represent the distribution of data points for participants that received placebo (red) or EPA (blue). The insert box in (A) highlights the few participants who were randomly assigned to placebo but displayed a high ΔEPA value. The dashed line in (A) represents the cut-off ΔEPA value (0.5 % points) used for the treatment-independent analysis of colorectal polyp outcomes according to the ΔEPA value during the first 6 mo of trial intervention. Abbreviation: HUFA, highly unsaturated fatty acid.

Participants who received EPA demonstrated a clear increase in RBC EPA content at V4 and V6 compared with placebo users, although there was considerable interindividual variability in response to EPA supplementation with clear overlap in ΔEPA values between EPA and placebo users (Figure 3A and Supplemental Figure 3). Similar results were obtained when participants who had stopped active IMP during the trial were excluded (Supplemental Figure 4). Interestingly, a few participants who had been randomly assigned to placebo displayed a change in RBC EPA concentration during trial participation that was similar to participants taking EPA (Figure 3A insert box). Analysis of the FFQ data for these participants did not suggest a significant increase in dietary omega-3 HUFA intake, and there was no concurrent increase in DHA concentration in these individuals (data not shown), which infers that these participants may have consumed an EPA-containing supplement, in addition to the trial IMP. A multivariate model was developed to identify factors that predicted the ΔEPA in the whole seAFOod trial cohort. The model included allocation to EPA, % compliance with capsules (placebo and EPA), allocation to aspirin, the formulation of EPA (FFA or TG), and female sex, as well as the baseline (V1) RBC EPA % content [21]. As expected, allocation to EPA treatment and the concentration of compliance with capsule intake were both positively associated with the ΔEPA value (Table 2). In addition, the baseline RBC % EPA value predicted the on-treatment change in EPA concentration (estimate −0.55; *P* < 0.0001). There was also a weak effect of the formulation of EPA taken by trial participants, with the EPA-TG formulation being associated with a lower ΔEPA value than EPA-FFA use (estimate −0.23; *P* = 0.036).

**TABLE 2:** Multivariate model of factors related to the change in % RBC EPA concentration measured after 6 mo of the seAFOod trial intervention period compared with the respective baseline value (ΔEPA).

ΔEPA	Effect estimate	95% confidence limits	t value	P value
Intercept	**−**0.04	**−**0.37, 0.30	**−**0.22	0.83
Allocation to EPA	1.32	1.06, 1.57	10.23	<0.0001
% capsule compliance	0.51	0.19, 0.84	3.07	0.002
Baseline (V1) RBC % EPA content	**−**0.55	**−**0.76, **−**0.33	**−**4.97	<0.0001
EPA formulation (TG)	**−**0.23	**−**0.44, **−**0.02	**−**2.11	0.036
Allocation to aspirin	**−**0.13	**−**0.39, 0.12	**−**1.01	0.093
Sex (female)	**−**0.04	**−**0.31, 0.23	**−**0.31	0.75

Abbreviations: RBC, red blood cell; TG, triglyceride.

Participants randomly assigned to EPA displayed an increase in RBC *n*–3DPA content, unlike placebo users, consistent with elongation of EPA to *n*–3DPA in some individuals (Figure 3B and Supplemental Figures 3 and 4). However, there was no evidence of conversion to DHA in EPA users, with overlap of ΔDHA values between EPA and placebo users (Figure 3C and Supplemental Figures 3 and 4).

### Analysis of seAFOod trial colorectal polyp outcomes according to the change in RBC EPA content during the intervention phase

**FIGURE 4:**
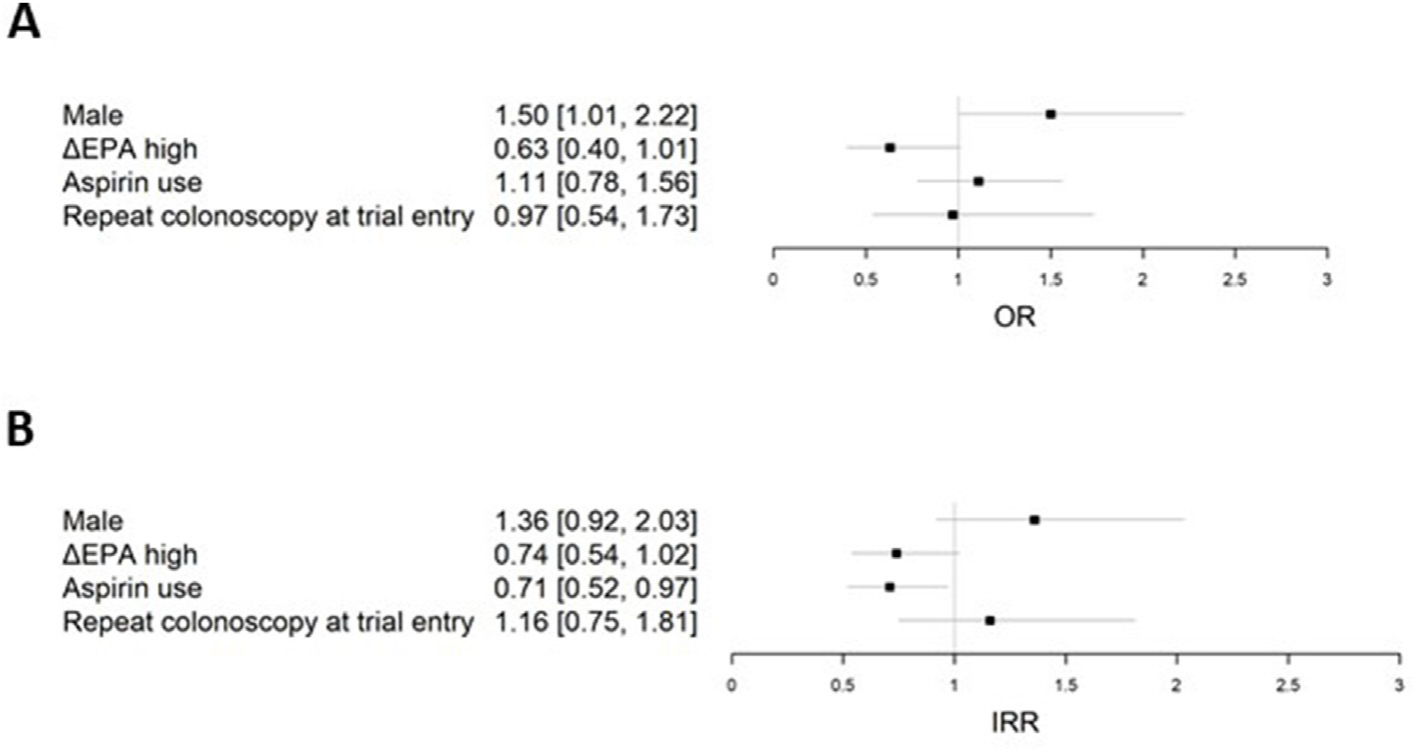
Multivariate models of (A) colorectal polyp detection rate, and (B) colorectal polyp number in seAFOod trial participants independent of treatment allocation to EPA or placebo. The change in RBC EPA concentration at 6 mo from the baseline concentration (ΔEPA_high_; defined as an increase in RBC% EPA concentration >0.5% points) was added to (A) logistic regression, and (B) negative binomial regression models, including sex, allocation to aspirin use, and repeat colonoscopy at baseline as covariables. Abbreviation: RBC, red blood cells.

Given the wide distribution of ΔEPA values in trial participants who received EPA treatment and the number of individuals allocated to placebo who displayed a ΔEPA concentration consistent with EPA supplementation (Figure 3A), we performed a treatment-independent analysis of colorectal polyp outcomes according to the ΔEPA value during treatment (V4). Based on a ΔEPA value of 0.5 percentage points, below which 91% of placebo users were situated (Figure 3A), there were 181 ΔEPA_high_ individuals (159 EPA, 22 placebo) and 289 ΔEPA_low_ participants (219 placebo, 70 EPA). ΔEPA_high_ was included as a covariate in the logistic regression and negative binomial regression models that had been used previously for analysis of colorectal PDR and number (Figure 4) [1,2,7,9]. Consistent with the primary and secondary outcomes of the seAFOod trial, aspirin use was associated with reduced colorectal polyp number, but not the PDR (Figure 4). Moreover, male sex predicted colorectal polyp recurrence in both models (Figure 4). Participants who displayed an increase in the RBC EPA concentration ≥0.5% points during the intervention phase of the trial (ΔEPA_high_; Figure 3), irrespective of whether they were allocated to active EPA capsules or placebo capsules, had lower colorectal polyp recurrence than ΔEPA_low_ individuals (Figure 4). The odds ratio for the PDR was 0.63 (95% CI: 0.40, 1.01), which was just above the nominal statistical significance threshold of 5% (*P* = 0.05). Participants with an increase in the RBC EPA concentration ≥0.5% points displayed a reduced colorectal polyp number at the end of the trial intervention period [incidence rate ratio 0.74 (95% CI: 0.54, 1.02)] compared with ΔEPA_low_ individuals, a difference which just missed statistical significance (*P* = 0.06; Figure 4).

## Discussion

Despite the presence of EPA and DHA in the diet, which could confound the outcomes in omega-3 HUFA intervention trials, to the best of our knowledge, no previous randomized intervention trial of supplemental EPA and/or DHA use has combined these 2 sources of omega-3 HUFAs for a secondary analysis of trial outcomes. Herein, we combined the estimated daily dietary intake of EPA+DHA during trial participation with compliance-adjusted supplemental EPA intake in order to determine the overall daily intake of EPA+DHA of each participant during the trial intervention period.

### Dietary omega-3 HUFA intake and RBC/tissue omega-3 concentrations

The relationship between dietary omega-3 HUFA intake and RBC membrane EPA+DHA content is well established [22]. By contrast, the relationship between dietary omega-3 HUFA intake and omega-3 HUFA concentrations in “target” colorectal mucosa in humans has received limited attention. Previously, we reported a weak but statistically significant association between the RBC EPA concentration and the corresponding rectal mucosal EPA concentration at the end of the intervention period (V6) in seAFOod trial participants [2]. Shen et al. [23] also reported a positive relationship between RBC and colonic mucosal omega-3 HUFA concentrations (% EPA+DPA+*n*–3DHA) in individuals who received a mixed omega-3 HUFA fish oil supplement for 12 wk. However, in the current analysis, we did not observe a clear relationship between dietary intake of omega-3 HUFAs and rectal mucosal omega-3 HUFA content in trial participants who received placebos only. This contrasts with data from an uninfected *Smad3*^-/-^ mice model, which demonstrated a strong correlation between RBC and colonic EPA+DHA concentrations across a range of 4 omega-3 HUFA-containing diets that contained increasing amounts of omega-3 HUFAs [24].

### Variability in omega-3 HUFA concentrations in seAFOod trial participants

We also report that, although dietary intake of EPA+DHA was well-balanced across the 4 treatment groups in the seAFOod trial, individual variability in dietary omega-3 HUFA intake led to considerable overlap in total EPA+DHA intake and blood/tissue omega-3 HUFA concentrations across groups that received either EPA or placebo. Overlap between EPA and placebo users was explained by a combination of high dietary intake of omega-3 HUFAs in some placebo users and poor compliance with EPA capsules in some participants allocated to EPA. Placebo group “contamination”—that is, unauthorized use of an alternative EPA-containing formulation by seAFOod trial participants—might have occurred but is likely to have been restricted to a handful of participants based on the small number of participants with a high ΔEPA value that were allocated to placebo capsules.

### The change in EPA concentration during the seAFOod trial and colorectal polyp outcomes

The large interindividual variability in circulating blood omega-3 HUFA concentrations in response to standardized dosing with purified omega-3 HUFA formulations or dietary omega-3 HUFA supplements has long been recognized [25,26]. The substantial overlap in RBC EPA concentrations in participants from EPA and placebo groups, related to differential dietary omega-3 HUFA intake and variable capsule compliance, prompted an intervention-independent analysis of the primary and secondary colorectal polyp outcomes of the seAFOod trial according to RBC EPA concentration rather than treatment (placebo compared with EPA) allocation.

Although the “omega-3 index” (the % EPA and DHA content in RBCs) has been proposed as a predictor of future cardiovascular disease risk [27], there is no accepted target EPA+DHA level in RBCs that might predict reduced cancer risk. Moreover, the distribution of individual on-treatment RBC EPA or EPA+DHA concentrations across the overall trial cohort did not justify the use of a particular RBC EPA value to dichotomize participants as either EPA_high_ or EPA_low_. Therefore, we used the individual change in EPA concentration between trial entry and after 6 mo of trial intervention as the outcome with which to determine colorectal polyp outcomes related to omega-3 HUFA status, irrespective of participant allocation to EPA or placebo.

Consistent with several other omega-3 HUFA intervention studies that have reported longitudinal measurements of RBC omega-3 HUFA concentrations, we found that participants with a low baseline RBC EPA concentration had a larger change in EPA concentration during the intervention phase [21]. Our finding also mirrored a secondary analysis of changes in plasma omega-3 HUFA and oxylipin concentrations associated with an EPA+DHA intervention in the VITAL trial, which reported that larger increases in EPA and DHA concentrations were observed in trial participants who had low fish intake (<1 serving per month) at baseline, compared with individuals that reported high fish intake (≥3.9 servings per week) at baseline [28]. We also observed a weak relationship between the size of the ΔEPA value and the type of EPA formulation that each participant received, with FFA use being associated with a larger increase in EPA concentration during trial treatment compared with EPA-TG use. This is consistent with several short-term dosing studies that have compared FFA and TG forms of omega-3 HUFA supplements [29].

The treatment-independent analysis of colorectal polyp outcomes uncovered a relationship between change in RBC EPA concentration and colorectal polyp risk measured as the PDR, which was a null endpoint in the primary analysis of the seAFOod trial, that just missed formal statistical significance. An increase in RBC EPA concentration during the trial, irrespective of the cause (trial treatment, “own use” omega-3 HUFA supplementation, and/or dietary omega-3 HUFA intake), was also associated with a trend toward reduced colorectal polyp number.

A similar post hoc approach to identify omega-3 HUFA “responders” has been taken in the randomized OMEGA-REMODEL trial of mixed omega-3 HUFAs in patients who had an acute myocardial infarction, in which Bernhard et al. [30] demonstrated that only individuals with an increase in omega-3 index >5% during the trial intervention period demonstrated a reduction in major adverse cardiovascular events during follow-up.

### HUFA degradation during RBC storage during the seAFOod trial

We do not believe that the small amount of HUFA degradation that likely occurred in RBC samples that were stored temporarily at temperatures >−80^o^C has confounded the analysis of the relationship between intake and RBC concentrations reported here or other seAFOod trial primary or secondary analyses that have compared trial treatment groups [1,2,9,10]. In the multivariate model, including factors that could explain differences in the baseline RBC EPA+DHA concentration in seAFOod trial participants, the interaction term for storage duration and temperature >−80^o^C suggested an extremely small effect size compared with dietary EPA+DHA intake. In the absence of a formal prospective time-course experiment mimicking temperature and aliquot size of seAFOod trial samples, formal comparison with other studies is not possible. However, the rate of HUFA loss in seAFOod trial RBC samples appears low compared with % reduction rates over 26 to 52 wk reported in other studies [6,[11], [12], [13], [14], [15]]. We postulate that the relatively large size of seAFOod trial RBC samples (thereby reducing the surface area to volume ratio) explains relatively low HUFA loss over time even at temperatures >−80^o^C [15].

We acknowledge several limitations of our study, including the inaccuracy of omega-3 HUFA intake measurements inherent in the use of a FFQ to measure dietary intake and capsule counting to determine IMP compliance [31]. We did not assess other sources of dietary omega-3 HUFAs, including nuts, seeds, and plant oils, which contain C18:3*n*–3 ALA that can be converted to EPA and DHA. It should also be borne in mind that we used our established LC-MS/MS method for relative quantification of HUFA concentrations rather than a more commonly used gas chromatographic technique. In addition, the seAFOod trial was relatively small (*n* = 707 randomly assigned participants who provided data), which predisposes to type 2 statistical error that may have been apparent in the treatment-independent analysis of colorectal polyp outcomes.

In conclusion, in a secondary analysis of the seAFOod trial, we highlight the large variability in RBC and rectal mucosal EPA concentrations in trial participants who received EPA 2000 mg free fatty acid, as well as considerable overlap in tissue EPA concentrations between individuals who were allocated to EPA or placebo. We describe a novel approach whereby we calculated total EPA and DHA intake of seAFOod trial participants as the sum of dietary intake and compliance-adjusted supplemental intake. This approach could be used to confirm the lack of confounding of clinical outcomes by other sources of omega-3 HUFAs in omega-3 HUFA intervention trials.

Our findings underscore the need for personalized diet and supplement interventions in colorectal cancer prevention, considering individual variability in diet, supplement compliance, and baseline omega-3 HUFA concentrations. Future research should aim to identify optimal target blood concentrations of EPA and DHA that predict reduced cancer risk and to understand the mechanisms governing the incorporation of omega-3 HUFAs into colorectal tissue and blood fractions.

## Authors’ contributions

The authors’ responsibilities were as follows – PML, EAW, MAH: conceived and designed the study; MAH: gained approvals for the study; GS, HFu, HFe, ADR, AD, PML, EAW, MAH: were involved in the acquisition, analysis, or interpretation of data; GS, HFu, MAH: drafting of the manuscript; EAW, CJR, LCB, PML, MAH: obtained funding for the study; and all the authors: contributed to the critical review and final approval of the manuscript; GS, HFu, HFe, AD, MAH: have accessed and verified the underlying data; and all authors: were responsible for the decision to submit the manuscript.

## Data sharing

The biomarker, as well as linked clinical and trial outcomes, datasets described in this report contain identifiable data, which are stored in a secure virtual research environment at the University of Leeds. Data will be anonymized and exported upon request to the Principal Investigator (Professor Hull) and the Study Sponsor (University of Leeds).

## Funding

This project (NIHR128210) was funded by the Efficacy and Mechanism Evaluation (EME) Programme, an MRC and NIHR partnership. The views expressed in this publication are those of the authors and not necessarily those of the MRC, NIHR or the Department of Health and Social Care. MAH and CJR are NIHR Senior Investigators. AD and MAH were supported by Cancer Research UK grant C23434/A24939.

## Conflicts of interest

The authors report no conflicts of interest.
