# Supplementary material for: The Relationship Between Dietary and Supplemental omega-3 Highly Unsaturated Fatty Acid Intake, Blood and Tissue omega-3 Highly Unsaturated Fatty Acid Concentrations, and Colorectal Polyp Recurrence: A Secondary Analysis of the seAFOod Polyp Prevention Trial
Source: J Nutr. 2024 Dec 31;155(2):549–58. doi: 10.1016/j.tjnut.2024.12.004 (PMC7617434; doi:10.1016/j.tjnut.2024.12.004)
Supplement: multimedia component 1 [file mmc1.pdf]

The relationship between dietary and supplemental *n*-3 HUFA intake, blood and tissue *n*-3 HUFA levels, and colorectal polyp recurrence: A secondary analysis of the seAFOod polyp prevention trial.

Sun G, *et al*.

Supplementary Data

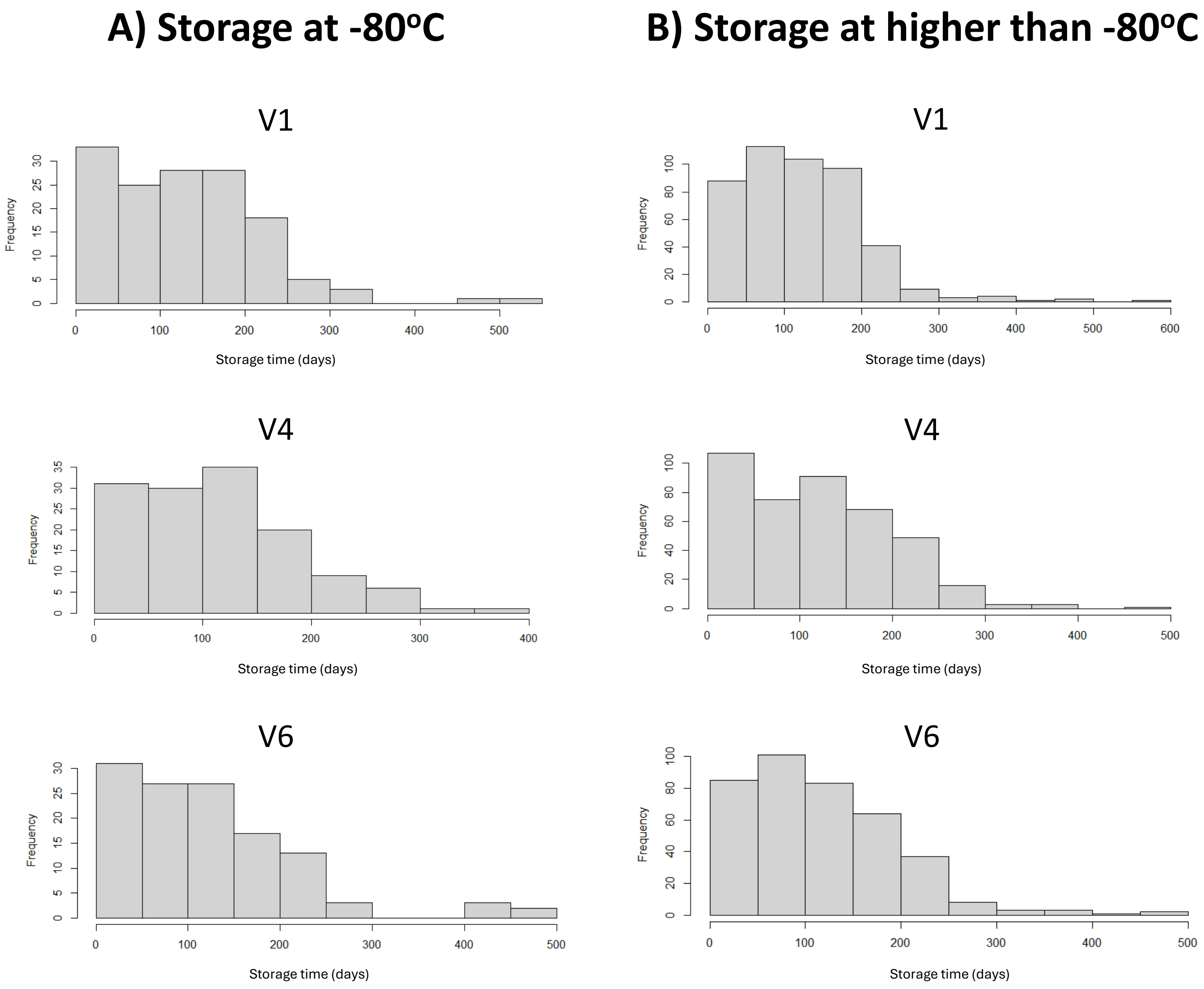

Supplementary Figure 1. The distribution of sample storage times at research sites before transfer to the -80°C central seAFOod trial biobank according to the duration of storage at trial research sites for samples stored at A) -80°C and B) any temperature higher than -80°C. Overall, 45% of samples were stored at research sites for less than 100 days at -80°C and 45% of samples were stored at research sites for less than 100 days at a temperature higher than -80°C .

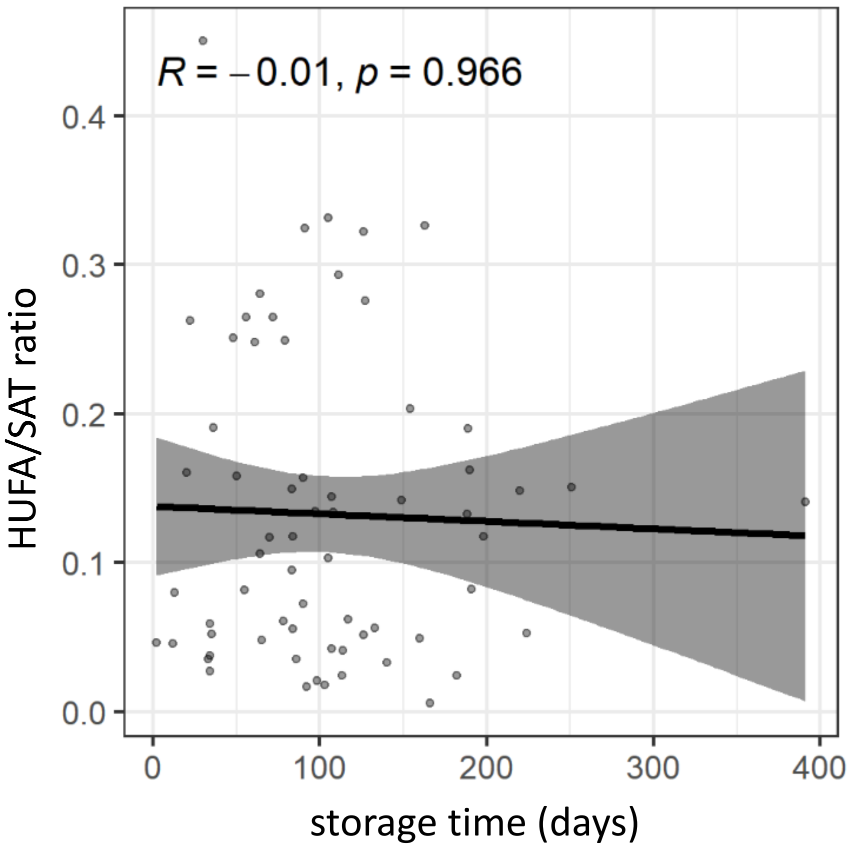

Supplementary Figure 2. The ratio of HUFAs to saturated fatty acids (HUFA/SAT ratio) in rectal mucosa from seAFOod trial participants allocated to placebos only (n=125) according to the duration of storage at trial research sites. Individual data points represent the HUFA/SAT ratio for the rectal mucosal sample obtained at colonoscopy at the end of the trial intervention period. Seventy-five (60%) rectal mucosal samples were stored at -20°C, seven (6%) samples were stored at a temperature between -21°C and -30°C, 17 (14%) samples were stored at a temperature between -31°C and -79°C and 26 (20%) samples were stored at -80°C.

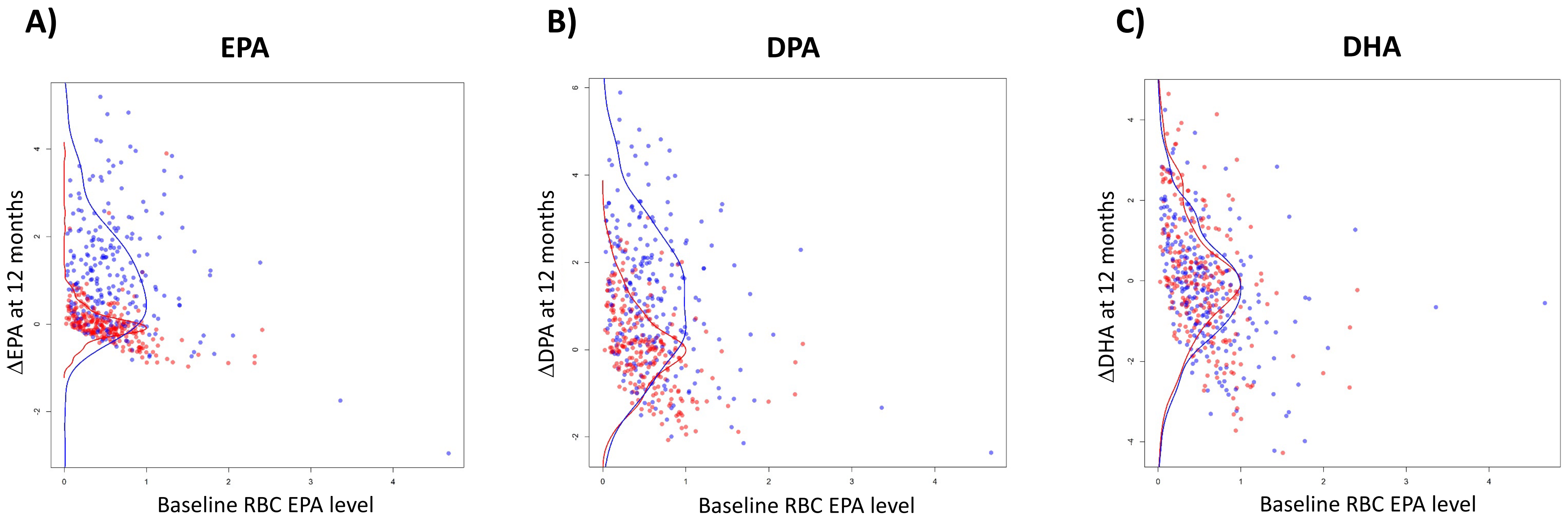

Supplementary Figure 3. The change in RBC levels of EPA, DPA and DHA at 12 months during seAFOod trial participation according to randomisation to EPA or placebo. The difference in omega-3 HUFA level after treatment for 12 months (V6) compared with baseline (V1) is plotted at individual participant-level according to the baseline EPA value for A) EPA, B) DPA and C) DHA. Summary curves represent the distribution of data points for participants that received placebo (red) or EPA (blue).

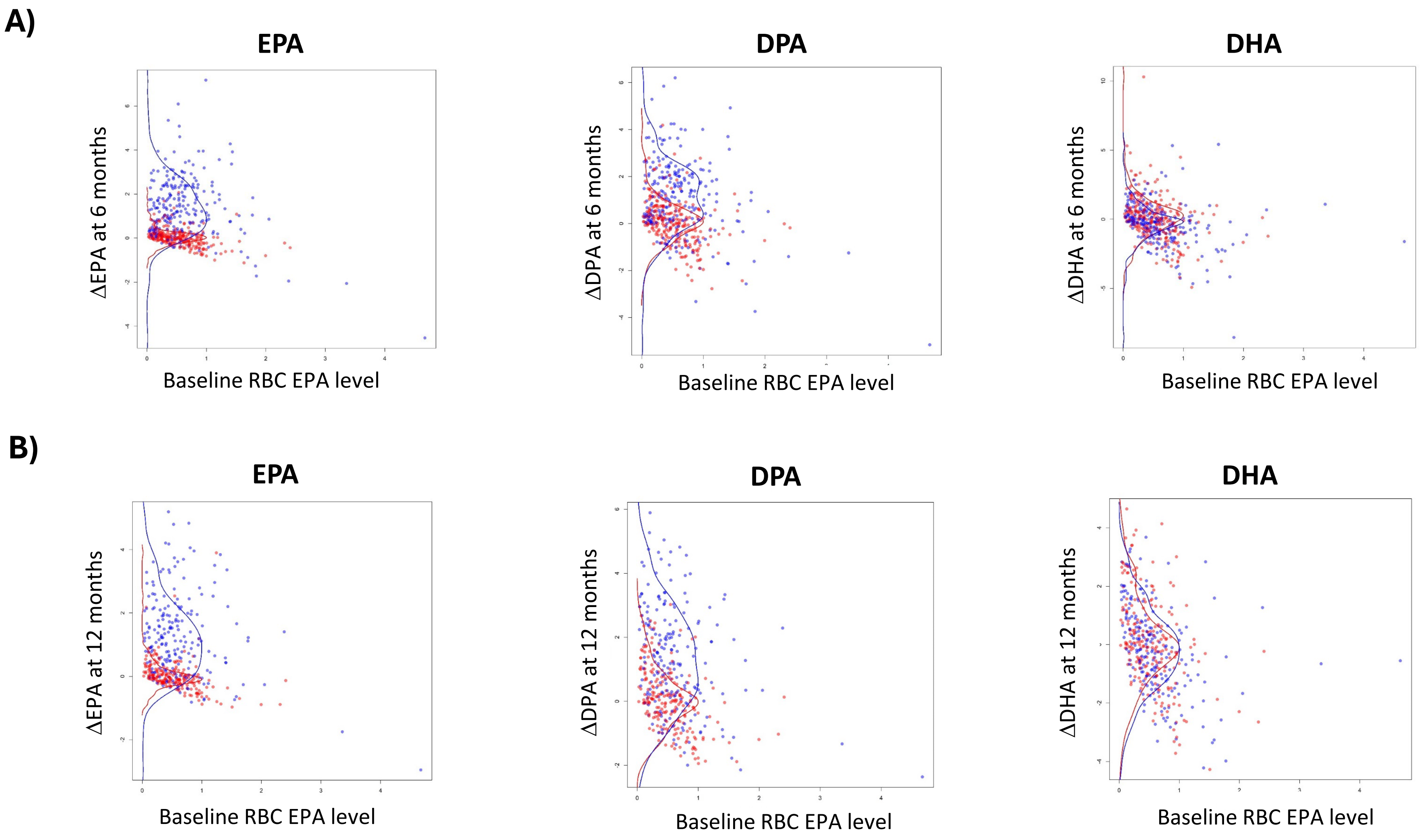

Supplementary Figure 4. The change in RBC levels of EPA, DPA and DHA during seAFOod trial participation according to ongoing use of EPA or placebo at either 6 or 12 months. The difference in omega-3 HUFA level after treatment for either (A) 6 or (B) 12 months compared with baseline (V1) is plotted at individual participant-level according to the baseline EPA value for EPA, DPA and DHA. Summary curves represent the distribution of data points for participants that received placebo (red) or EPA (blue).

Supplementary Table 1. The number of seAFOod trial participants providing data for RBC and rectal mucosal omega-3 PUFA levels and a completed food frequency questionnaire

	Placebos only	EPA	Aspirin	EPA & Aspirin	Total
baseline RBC HUFAs (V1)	156	155	159	154	624
Mid-treatment RBC HUFAs (V4)	146	131	142	141	560
Trial exit RBC HUFAs (V6)	135	128	131	128	522
Rectal mucosal HUFAs (V6)	136	122	134	127	519
Baseline FFQ (V1)	162	164	161	162	649
Trial exit FFQ (V6)	138	129	134	126	527

EPA, eicosapentaenoic acid; FFQ, food frequency questionnaire; HUFA, highly unsaturated fatty acid; RBC, red blood cell; V1, visit 1 (baseline, before intervention; V4, visit 4 (mid-treatment at 6 months); V6, visit 6 (end of treatment/exit colonoscopy at 12 months)

Supplementary Table 2. The number (%) of seAFOod trial participant red blood cell samples stored at either minus 80°C or a temperature greater than minus 80°C at research sites according to treatment allocation.

	Storage temp	Placebos only	EPA	Aspirin	EPA & Aspirin	Total	P value[1]
**visit 1**	minus 80°C	35 (23)	39 (26)	31 (20)	37 (25)	142 (23)	0.70
	>minus 80°C	115 (77)	112 (74)	122 (80)	114 (75)	463 (77)	
**visit 4**	minus 80°C	31 (22)	32 (25)	37 (27)	33 (24)	133 (24)	0.78
	>minus 80°C	111 (78)	96 (75)	100 (73)	106 (76)	413 (76)	
**visit 6**	minus 80°C	30 (23)	31 (25)	33 (26)	29 (23)	123 (24)	0.96
	>minus 80°C	101 (77)	95 (75)	96 (74)	95 (77)	387 (76)	

[1]Pearson's χ^2 test comparing the proportions of samples in the two storage temperature categories across the four treatment groups.

Supplementary Table 3. The relationship between clinical characteristics of seAFOod trial participants and red blood cell EPA and DHA levels prior to intervention

	n	%EPA[1] median (IQR[2])	%EPA[1] *P*	%DHA[1] median (IQR)	%DHA[1] *P*	%EPA+%DHA[1] median (IQR)	*P*
Sex			**0.066**[3]		0.16		0.13
Male	497	0.46 (0.26-0.71)		1.89 (1.01-2.88)		2.41 (1.31-3.55)	
Female	127	0.54 (0.29-0.80)		2.06 (1.30-3.08)		2.67 (1.57-3.83)	
Body Mass Index			**<0.001**		**0.009**		**0.002**
Underweight (<18.5 Kg/m^2)	3	0.29 (0.28-0.33)		1.62 (1.06-1.67)		1.91 (1.34-2.00)	
Normal (≥18.5 and <25 Kg/m^2)	113	0.54 (0.33-0.82)		2.22 (1.34-3.13)		2.90 (1.70-3.99)	
Overweight (≥25 and <30 Kg/m^2)	265	0.51 (0.31-0.77)		2.03 (1.06-3.03)		2.59 (1.37-3.78)	
Obese (≥30 Kg/m^2)	240	0.39 (0.22-0.63)		1.67 (0.89-2.62)		2.13 (1.15-3.26)	
Diabetes			0.44		0.93		0.84
No	558	0.47 (0.26-0.76)		1.90 (1.01-2.95)		2.44 (1.31-3.69)	
Yes	66	0.44 (0.27-0.62)		2.01 (1.32-2.52)		2.46 (1.56-3.16)	
Tobacco smoking			0.22		0.06		0.07
Never	225	0.48 (0.29-0.78)		2.08 (1.06-2.90)		2.63 (1.43-3.61)	
Ever	307	0.47 (0.24-0.76)		1.99 (1.09-3.02)		2.50 (1.39-3.85)	
Current	92	0.41 (0.26-0.62)		1.60 (0.84-2.55)		2.11 (1.18-3.11)	
Alcohol intake			**0.026**		0.56		0.41
None	98	0.40 (0.23-0.68)		1.68 (0.84-2.75)		2.15 (1.03-3.52)	
1-7 units/week	213	0.45 (0.25-0.71)		1.96 (1.04-2.96)		2.39 (1.37-3.61)	
8-21 units/week	186	0.46 (0.26-0.73)		1.89 (1.10-2.88)		2.48 (1.38-3.53)	
≥22 units/week	126	0.53 (0.34-0.85)		2.08 (1.29-3.04)		2.63 (1.62-3.91)	

[1]The % of total measured fatty acids in red blood cell membranes

[2]IQR, inter-quartile range

[3]P values denoting statistical significance (<0.05) are in bold type

Supplementary Table 4. Rectal mucosal EPA and DHA levels according to clinical characteristics in individuals receiving placebos only during the seAFOod trial.

		%EPA[1]		%DHA[1]		%EPA+%DHA[1]	
	**n**	**median (IQR[2])**	***P***	**median (IQR)**	***P***	**median (IQR)**	***P***
Sex			0.44		0.13		0.21
Male	108	0.45 (0.25-0.63)		0.93 (0.64-1.31)		1.39 (0.89-2.03)	
Female	28	0.51 (0.30-0.68)		1.18 (0.73-1.68)		1.73 (1.13-2.38)	
Body mass Index[3]			0.67		0.48		0.43
Normal (≥18.5 and <25 Kg/m^2)	26	0.42 (0.25-0.62)		0.85 (0.55-1.23)		1.25 (0.79-1.82)	
Overweight (≥25 and <30 Kg/m^2)	57	0.46 (0.28-0.69)		0.94 (0.70-1.44)		1.39 (0.97-2.19)	
Obese (≥30 Kg/m^2)	52	0.48 (0.29-0.63)		1.01 (0.62-1.45)		1.53 (0.85-2.10)	
Diabetes			0.13		0.36		0.20
No	116	0.43 (0.25-0.63)		0.94 (0.64-1.31)		1.37 (0.89-1.98)	
Yes	20	0.58 (0.38-0.72)		1.09 (0.80-1.53)		1.88 (1.20-2.26)	
Tobacco smoking			0.92		0.99		0.99
Never	48	0.52 (0.23-0.69)		1.00 (0.58-1.46)		1.53 (0.83-2.10)	
Ever	64	0.45 (0.29-0.63)		0.93 (0.70-1.41)		1.36 (0.98-2.09)	
Current	24	0.48 (0.30-0.60)		0.98 (0.65-1.30)		1.44 (0.99-1.84)	
Alcohol intake			0.16		0.34		0.27
None	24	0.34 (0.25-0.52)		0.86 (0.56-1.36)		1.19 (0.81-1.88)	
1-7 units/week	41	0.40 (0.23-0.86)		0.94 (0.51-1.67)		1.45 (0.75-2.77)	
8-21 units/week	42	0.48 (0.25-0.59)		0.97 (0.68-1.27)		1.42 (0.90-1.86)	
≥22 units/week	29	0.52 (0.41-0.64)		1.11 (0.87-1.51)		1.59 (1.24-2.19)	

[1]The % of total measured fatty acids in red blood cell membranes

[2]IQR, inter-quartile range

[3]missing data on one participant
